# SARS-CoV-2: A Glance at the Innate Immune Response Elicited by Infection and Vaccination

**DOI:** 10.3390/antib13010013

**Published:** 2024-02-08

**Authors:** Nicola Manfrini, Samuele Notarbartolo, Renata Grifantini, Elisa Pesce

**Affiliations:** 1 INGM, Istituto Nazionale Genetica Molecolare “Romeo ed Enrica Invernizzi”, 20122 Milan, Italy; 2 Department of Biosciences, University of Milan, 20133 Milan, Italy; 3 Infectious Diseases Unit, Foundation IRCCS Ca’ Granda Ospedale Maggiore Policlinico, 20122 Milan, Italy; 4 CheckmAb Srl, 20122 Milan, Italy; 5 Department of Clinical Sciences and Community Health, University of Milan, 20122 Milan, Italy

**Keywords:** COVID-19, SARS-CoV-2, innate immunity, vaccines, immune response, human immunology

## Abstract

The COVID-19 pandemic caused by Severe Acute Respiratory Syndrome Coronavirus 2 (SARS-CoV-2) has led to almost seven million deaths worldwide. SARS-CoV-2 causes infection through respiratory transmission and can occur either without any symptoms or with clinical manifestations which can be mild, severe or, in some cases, even fatal. Innate immunity provides the initial defense against the virus by sensing pathogen-associated molecular patterns and triggering signaling pathways that activate the antiviral and inflammatory responses, which limit viral replication and help the identification and removal of infected cells. However, temporally dysregulated and excessive activation of the innate immune response is deleterious for the host and associates with severe COVID-19. In addition to its defensive role, innate immunity is pivotal in priming the adaptive immune response and polarizing its effector function. This capacity is relevant in the context of both SARS-CoV-2 natural infection and COVID-19 vaccination. Here, we provide an overview of the current knowledge of the innate immune responses to SARS-CoV-2 infection and vaccination.

## 1. Introduction

Coronavirus Disease 2019 (COVID-19) is caused by Severe Acute Respiratory Syndrome Coronavirus 2 (SARS-CoV-2) and was declared a pandemic by the World Health Organization (WHO) in March 2020 (https://covid19.who.int, accessed on 1 December 2023) [1].

The spectrum of clinical symptoms observed in SARS-CoV-2-infected individuals can vary from positive testing (without any evident clinical manifestation) to mild/moderate symptoms (characterized by fever, cough, sore throat, malaise, headache, myalgia, nausea/vomiting, diarrhea, anosmia, and ageusia) to severe disease (defined by SpO_2_ levels below 94%, respiratory frequency of up to 30 breaths per minute, or inflammatory pulmonary infiltrate in more than 50% of the organ) (https://www.covid19treatmentguidelines.nih.gov/overview/clinical-spectrum/, accessed on 1 December 2023).

Critical illness is marked by acute respiratory distress, multi-organ failure, septic shock, and, in the worst scenario, death (https://www.covid19treatmentguidelines.nih.gov/overview/clinical-spectrum/, accessed on 1 December 2023). During the first waves of the pandemic, approximately 10–15% of confirmed cases were estimated to progress into a severe form of the disease, requiring hospitalization, with a higher incidence rate among the elderly. Specific comorbidities, such as cardiovascular and metabolic diseases, as well as other less-characterized factors (e.g., co-infections) are also associated with COVID-19 severity [2].

SARS-CoV-2 possesses an enveloped structure housing a genome of approximately 30 kb, characterized by positive-sense, single-stranded RNA (ssRNA) [3,4]. The 3′-end of the viral genome encodes for four structural components: the spike (S), envelope (E), membrane (M), and nucleocapsid (N) proteins [5]. The S protein is a glycoprotein that mediates viral particle entry by attaching to and fusing with the host cell membrane. The S protein is present in multiple copies in the virion membrane which are assembled into homotrimers [6]. It is cleaved into the S1 and S2 subunits by proprotein convertases, such as furin, which are expressed by infected cells, giving rise to a mature S protein that is composed of the two non-covalently associated subunits [7]. The S1 subunit consists of four major structural domains, which include the amino-terminal domain (NTD), the receptor-binding domain (RBD), and two carboxy-terminal domains (CTDs) that protect the inner S2 subunit. The S1 subunit binds the host cell receptor Angiotensin-Converting Enzyme 2 (ACE2) through the RBD, while the S2 subunit anchors the S protein to the membrane [8].

The ACE2 receptor is highly expressed on the surface of cells in many tissues and organs, especially in the mucosa of the respiratory and digestive tract and in the myocardium [9], making these tissues particularly susceptible to SARS-CoV-2 infection.

Efficient engagement of the ACE2 receptor by SARS-CoV-2 necessitates auxiliary co-factors such as SLC1A5 [10] or co-receptors like vimentin [11] and the additional cleavage of an extra site within the S2 subunit, referred to as the ‘S2 site,’ which is facilitated either by Transmembrane Serine Protease 2 (TMPRSS2) on the cell surface or cathepsin L in the endosomal compartment [12]. This cleavage induces a significant conformational alteration in both the S1 and S2 subunits, promoting fusion between the virus and host cell membranes and the creation of a pore. This pore enables the viral genome to enter the host cell, initiating the replication process [13].

The initial defense against pathogens is provided by the innate immune system. It works by directly counteracting the infection and by inducing and sustaining an optimal adaptive immune response.

Innate immunity is a defense mechanism with a limited specificity that is usually activated within hours after pathogen exposure and does not generate immunological memory.

The innate immune response can be divided into 1) the immediate innate immunity and 2) the induced innate immunity. The first is very rapidly activated after infection (0–4 h) and relies on the action of preformed soluble antimicrobial molecules, including antimicrobial enzymes and complement system proteins. The second begins later (4 to 96 h post-infection), involves the activation and the recruitment of defense cells, and lasts a few days. Innate immune cells include phagocytic cells, like neutrophils, monocytes, and macrophages, and natural killer cells, whose function is to destroy either the pathogen or the pathogen-infected cell [14].

**Figure 1:**
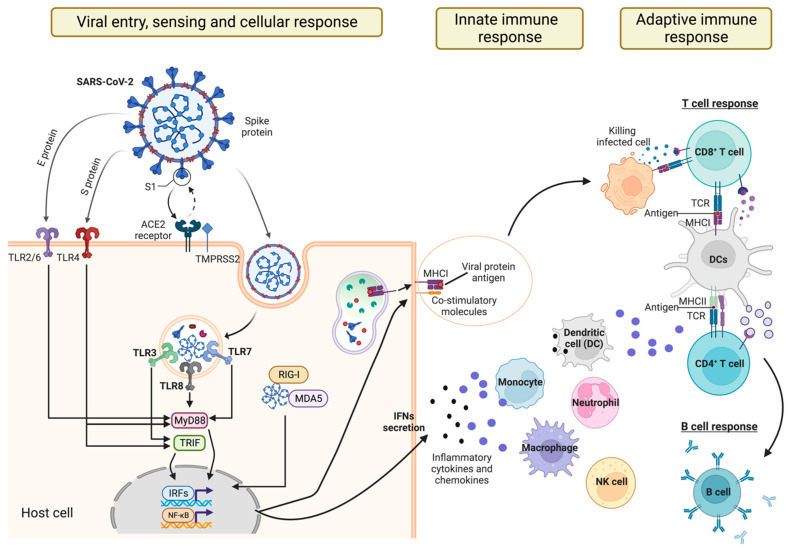
**Schematic representation of signaling pathways and immune responses induced by SARS-CoV-2 infection.** Before entering the host cell, SARS-CoV-2 is detected on the cell surface by specific TLRs capable of recognizing the E and S proteins (namely TLR2/6 and TLR4). Following host cell entry, the SARS-CoV-2 virus is recognized by RNA sensors present in both the cytosol and endosomes (RLRs and TLRs). Intracellular TLRs can detect endosomal ssRNA (TLR7/8) or dsRNA (TLR3), while RIG-I and MDA5 play a role in sensing viral RNAs within the cytoplasm. Upon recognition, these sensors recruit the signal adapter molecules MyD88 and TRIF, inducing downstream signaling. This leads to the activation of NF-κB and IRF transcription factors and the production of pro-inflammatory cytokines (e.g., IL6 and TNF-α) and type I IFNs (IFN-α and IFN-β). Viral protein antigens are also processed and presented on MHCs to T cells in the presence of co-stimulatory molecules. Cytokines and chemokines released by infected cells modulate the cellular innate response, favoring the infiltration of monocytes and neutrophils. Activated dendritic cells, instead, present the antigens to CD4^+^ T cells, which secrete cytokines favoring the production of immunoglobulins by mature B cells, and CD8^+^ T cells that directly kill infected cells. (This figure was created with BioRender.com). RLRs: retinoic acid-inducible gene I (RIG-I)-like receptors; TLRs: oll-like receptors; NF-κB: Nuclear Factor kappa B; MAPKs: mitogen-activated protein kinases; IRFs: interferon regulatory factors; MDA5: melanoma differentiation-associated gene 5.

During a viral infection, upon pathogen recognition, innate immune signaling is activated to establish an antiviral state that limits viral replication within the cell and reduces the susceptibility of surrounding cells to the infection. This signaling is mediated by the production and release of molecules such as interferons (IFNs), cytokines, chemokines, and surfactant proteins. These signaling molecules also play a role in triggering and drawing immune cells like neutrophils, macrophages, and natural killer cells to the infection site. There, these cells cooperate to clear the pathogen (Figure 1). In addition, the innate immune system plays a key role in the development of the adaptive immune response, primarily through dendritic cells (DCs). DCs are professional antigen-presenting cells (APCs) that present pathogen-derived antigens to naïve T cells, inducing the activation and proliferation of antigen-specific T cells and their differentiation into effector cells (Figure 1). By providing co-stimulatory signals and producing cytokines, DCs also polarize the effector function of T cells and optimize the generation of long-lived memory T cells, which provide enhanced protection upon re-exposure to the same pathogen [15].

In this review, we describe the means through which the innate immune system (1) senses and reacts to SARS-CoV-2 infection and (2) cooperates with the adaptive immune system to generate protective immunity upon vaccination.

## 2. Sensing SARS-CoV-2 Infection

The innate immune system detects the presence of pathogens by recognizing different molecules that are specifically expressed by microbes and collectively called pathogen-associated molecular patterns (PAMPs). The recognition of PAMPs is mediated by specific receptors named pattern-recognition receptors (PRRs) [16,17]. The binding of PAMPs to PRRs initiates the production of cytokines, chemokines, and adhesion molecules by innate immune cells, aiming to restrict infection, promote pathogen clearance, and, if the infection is not eradicated, activate the adaptive immune response [18].

Among the PRRs, SARS-CoV-2 is identified by and triggers specific toll-like receptors (TLRs), retinoic acid-inducible gene I (RIG-I)-like receptors (RLRs), nucleotide-binding oligomerization domain (NOD)-like receptors (NLRs), and the inflammasome [19] (Figure 1).

### 2.1. Recognition of SARS-CoV-2 by TLRs

The TLR family comprises ten members (TLR1-TLR10) in humans. Some TLRs are present on the cell surface and mainly participate in extracellular pathogen recognition, while others are found inside the cell, in endosomes or endolysosomes, and are engaged during infections by intracellular pathogens [20,21,22].

During a viral infection, after binding viral PAMPs, activated TLRs use two main adaptor molecules to transduce received signals: the Myeloid differentiation primary response 88 (MyD88) and the TIR domain-containing adapter-inducing interferon-β (TRIF) protein [23]. The MyD88-dependent pathway mainly activates the Nuclear Factors kappa-B (NF-κB) and the Activator Protein (AP-1) transcription factors and leads to transcriptional activation of pro-inflammatory cytokines. TRIF, instead, activates the Interferon Regulatory Factors 3 and 7 (IRF3 and IRF7) to produce type-I IFNs and mount an antiviral response and NF-κB to make pro-inflammatory cytokines, such as interleukin-1 (IL-1), interleukin-6 (IL-6), tumor necrosis factor-α (TNF-α), and interleukin-12 (IL-12) [23]. Depending on the input signal, such as when activated downstream of TRL7 and TLR9 stimulation, MyD88 can also activate IRFs and contribute to the antiviral response.

At least six TLRs have been associated with SARS-CoV-2 virus identification, i.e., TLR2, TLR3, TLR4, TLR7, TLR8, and TLR9 [24].

TLR2 and TLR4 are cell membrane-bound and can recognize structural and nonstructural proteins exposed on the surface of the virus [25]. Specifically, TLR2 recognizes SARS-CoV-2 E protein [26], while TLR4 recognizes the S1 subunit [27,28] (Figure 1). The engagement of these two TLRs triggers the production of inflammatory cytokines.

An involvement of TLR2 in SARS-CoV-2 recognition was originally predicted by Jung and colleagues through a computational study, based on single-cell sequencing data, aimed at defining inflammation modulators in patients with COVID-19 [29]. This prediction was later validated by Zheng and colleagues [26], who found that (1) TLR2 inhibition reduced inflammatory cytokine production and improved the survival of SARS-CoV-2-infected K18-hACE2 transgenic mice, and (2) SARS-CoV-2 E protein-induced pro-inflammatory signaling and cytokine production were weakened in human macrophages treated with a TLR2 inhibitor.

The activation of TLR4 with S1 stimulates the synthesis of pro-inflammatory cytokines (IL-1β and IL-18) which serve in the early stages of inflammasome formation whose activation results in IL-1 production and the secretion of the pleiotropic inflammatory mediator IL-6 by immune cells [30,31,32].

Among intracellular TLRs, TLR3 specifically senses viral dsRNA and signals through TRIF to activate a type-I IFN-dependent antiviral response. Like other positive-sense ssRNA viruses, SARS-CoV-2 produces double-stranded RNA (dsRNA) early during the infection cycle as a result of genome replication and mRNA transcription. Involvement of TLR3 in SARS-CoV-2 innate immune responses was demonstrated by Zhao et al., who showed that a functional TLR3 signaling pathway is required to reduce SARS-CoV-2 burden in human alveolar epithelial cells [33].

TLR7 and TLR8, instead, recognize ssRNA. After macrophage phagocyte SARS-CoV-2, the genomic ssRNA is discharged by virions and can be directly recognized by TLR7/TLR8 (Figure 1). Although TLR7 can activate both NF-κB and IRFs, in turn inducing inflammatory and antiviral responses, during SARS-CoV-2 lung infection, TLR7 has been shown to mainly activate the NF-κB pathway contributing to the overt production of pro-inflammatory cytokines in patients with severe COVID-19 [32,34]. TLR9 was also shown to be involved in the immune responses upon SARS-CoV-2 infection but data remain scanty.

### 2.2. Recognition of SARS-CoV-2 by RLRs

The Retinoic acid-inducible gene I (RIG-I)-like receptor (RLR) family is composed of cytoplasmic RNA helicases whose role is to sense non-self RNA [35]. RLRs include three homologous members: RIG-I, Melanoma Differentiation-Associated gene 5 (MDA5), and Laboratory of Genetics and Physiology 2 (LGP2) [36,37]. RLRs typically remain dormant in uninfected cells but become activated in the presence of viral RNA. During SARS-CoV-2 infection, RIG-I and MDA5 detect dsRNA viral genome intermediates (Figure 1) and repress viral replication [38,39,40]. RLRs are typically activated via interaction with the Mitochondrial Antiviral-Signaling (MAVS) protein to trigger type-I and type-III IFNs signaling pathways [41,42,43,44]. In the context of SARS-CoV-2 infection, Yang et al. [45] showed that a deficiency in MDA5, RIG-I, or MAVS augmented virus replication. Accordingly, during infection, efficient SARS-CoV-2 replication requires the hampering of RLR signaling via its papain-like protease to suppress the production of IFNs and the function of components of the RLR receptor signaling pathway, including RIG-I, MAVS, TBK1, TRAF3, TRAF6, and IRF3 [46,47]. Notably, children exhibit elevated basal expression levels of RIG-1 and MDA5 in upper airway epithelial cells. This results in a more vigorous and prompt initial antiviral response to SARS-CoV-2 when compared to adults [48] and may explain, at least in part, the lower incidence of COVID-19 in the pediatric population. In contrast, the reduced activity of type-I IFNs in the elderly due to the presence of neutralizing autoantibodies underlies the higher susceptibility of people older than 65 years of age to severe COVID-19 [49].

The role of RLR signaling in controlling SARS-CoV-2 infection is not limited to the induction of type-I IFN production. A recent study by Yamada et al. [50] showed that RIG-I can detect the 3′ untranslated region of the SARS-CoV-2 genome using its helicase domains, and not the more traditional C-terminal region. This interaction was shown to directly interfere with the first RNA polymerase-dependent replication step of SARS-CoV-2 in a type I/II IFN-independent manner. Moreover, Kouwaki and colleagues [51] found that RIG-I and MDA5 induce antiviral states by increasing the expression of inflammatory cytokines, such as CCL5 and CXCL10, in addition to type-I IFNs. A different study by Yin and colleagues [40] found, instead, no impact on IFN-β expression when RIG-I was silenced in Calu-3 cells, highlighting the complexity of this regulation and the uncertainties that still revolve around RIG-I mode of functioning upon SARS-CoV-2 infection.

### 2.3. Recognition of SARS-CoV-2 by NLRs

Among the inflammasomes responding to RNA viruses, the NLRP3 inflammasome is the best characterized [52]. NLRP3 activation is triggered in response to microbial infection via PAMPs or upon host cell damage, through damage-associated molecular patterns (DAMPs) signaling [53]. The NLRP3 inflammasome is composed of the NLRP3 receptor, the apoptosis-associated speck-like protein containing a caspase activation and recruitment domain (ASC) adaptor molecule and caspase-1 [54]. The activation of caspase-1 promotes the maturation of IL-1β and IL-18 pro-inflammatory cytokines and the cleavage of gasdermin (GSDM) D, a pore-forming protein that triggers pyroptosis, a type of inflammatory programmed cell death [55].

SARS-CoV-2 infection triggers the NLRP3 inflammasome, which contributes to the antiviral immune response. As described before, the SARS-CoV-2 E protein was demonstrated to elicit the activation of TLR2, which in turn upregulates both *NLRP3* and *IL1B* mRNA levels in macrophages [26]. Activation of the inflammasome can be further enhanced by GU-rich ssRNA processed from the SARS-CoV-2 genome. The involvement of the NLRP3 inflammasome in the immune response to SARS-CoV-2 was confirmed by the fact that the SARS-CoV-2 N protein interacts with NLRP3 and promotes binding with ASC, in turn facilitating the assembly of the inflammasome [56]. Moreover, human primary monocytes infected in vitro with SARS-CoV-2 showed NLRP3-dependent caspase-1 activation, GSDMD cleavage, and IL-1β production [57]. Lung tissues from patients with COVID-19 were shown to contain NLRP3 and ASC puncta, confirming that the NLRP3 inflammasome is indeed activated in infected patients with acute lung injury [58]. PAMPs derived from SARS-CoV, including those deriving from the accessory proteins ORF3a, ORF8b, and viral RNA, can promote the activity of the NLRP3 inflammasome [59], indicating that inflammasome activation is possibly a feature that is common with other Coronaviruses.

Although an efficient activation of different PRRs is fundamental for an effective immune response against SARS-CoV-2, their over-activation may have deleterious effects since excessive inflammation can lead to cell or tissue damage. This occurs, for example, when viral PAMPs and DAMPs released from lysed cells accumulate and over-stimulate TLRs and NLRs signaling, triggering the production of excessive amounts of pro-inflammatory cytokines and chemokines [24]. TLR4, in particular, is thought to have an important function in the pathogenesis of COVID-19 by triggering aberrant hyperinflammation.

A study by Hadjadj J. et al. [31] revealed that severe COVID-19 is associated with an impaired type-I IFN response and elevated production of TNF-α and IL-6, which is partly driven by NF-κB.

These data are consistent with the idea that a defective type-I IFN-dependent antiviral response leads to an inefficient control of viral replication resulting, in a few days, in the accumulation of viral particles and damaged cells. When overcoming a certain threshold, these PAMPs and DAMPs strongly stimulate an emergency inflammatory response that is aimed at controlling the infection but, in fact, results in additional tissue damage.

In conclusion, if inflammation is not properly controlled, it may result in a cytokine-storm-like syndrome that exacerbates COVID-19 symptoms and is frequently observed in patients with severe disease [60].

## 3. Innate Cell Responses to SARS-CoV-2 Infection

### 3.1. Macrophages, Monocytes, and SARS-CoV-2

Monocytes and macrophages are critical in providing rapid responses to pathogens during acute infections. They are essential for the recruitment of leukocytes, for regulating inflammation, and can function as APCs.

Under homeostatic conditions, macrophages are the most abundant immune cell type present in the lungs, the primary location of SARS-CoV-2 infection. Macrophages can be divided into two categories according to their location: interstitial macrophages (IMs), which reside in the parenchyma of the lungs between the microvascular endothelium and the alveolar epithelium, and alveolar macrophages (AMs), which are usually located in the alveoli in close contact with type I and type II alveolar epithelial cells [61]. AMs stand as the initial line of defense against pathogens infiltrating the respiratory system.

Following infection, TLRs expressed by AMs actively detect the N and S proteins of SARS-CoV-2, promoting their polarization into the M1 phenotype [62,63]. Once activated, AMs release pro-inflammatory cytokines (IL-1β and IL6) and chemokines (CXCL10), resulting in the recruitment and activation of other immune effector cells [64]. Among the recruited cells, monocytes can differentiate into either DCs or macrophages. Coherently, with recruitment into the infected tissue, a decrease in the frequency of circulating monocytes is found in patients with COVID-19, followed by a gradual increase after the end of the infection [65,66].

Monocyte-derived DCs and macrophages serve to enhance the immune response by presenting the viral antigen to T cells and amplifying inflammation [67]. They also contribute to tissue remodeling and repair and the termination of the effector response through the production of anti-inflammatory cytokines [68].

Interestingly, macrophages found close to the areas of damaged endothelial cells produce high amounts of type-I IFN, possibly due to the activation of the STimulator of Interferon Genes (STING) pathway [69]. SARS-CoV-2 infection induces mitochondrial damage in epithelial cells, leading to the accumulation of mitochondrial DNA (mtDNA) in the cytosol. This surplus of mtDNA triggers an excessive activation of STING, resulting in the demise of epithelial cells. Upon engulfing with cellular debris, macrophages sense mtDNA, activate the cGAS-STING signaling pathway, and secrete type-I IFN, eliciting a potent inflammatory response. These dysregulated cytokine responses propel immunopathology, contributing to tissue damage and pathology.

A study by Domizio et al. delved into the potential impact of inhibiting STING upon SARS-CoV-2 infection using a K18-hACE2 transgenic mouse model. These mice were treated with H-151, a small-molecule STING inhibitor, and the histological examination of the lungs showed a significant reduction of inflammation. Moreover, they found that the levels of pro-inflammatory genes (like *Il-6*), chemokines (like *Ccl2* and *Ccl3*), and markers of lung injury (e.g., *F3*) were also lower in mice treated with the STING inhibitor. Collectively, the data underscore that STING critically contributes to amplifying inflammatory responses during the later stages of infection [70,71].

SARS-CoV-2 was shown to also regulate monocyte and macrophage function by altering their antigen presentation capabilities, for instance, by down-modulating Human Leukocyte Antigen (HLA) expression [72]. Gatti et al. indeed found that individuals experiencing severe COVID-19 showed reduced HLA-DR expression on monocytes [73]. This decrease in HLA-DR expression is likely due to the antagonistic effects of IL-6, leading to immune suppression during SARS-CoV-2 infection [74].

### 3.2. Dendritic Cells and SARS-CoV-2

DCs are professional APCs playing a crucial role in the activation of antigen-specific T cells and the polarization of their effector function. Among DC subsets, plasmacytoid DCs (pDCs) are specialized in antiviral immunity through the production of large amounts of type-I IFN [75]. Circulating pDCs have been shown to be reduced in COVID-19 [76].

Saichi and colleagues observed a heightened expression of pro-apoptotic molecules in pDCs derived from patients affected by severe COVID-19, suggesting that pDCs in these patients have an increased rate of cell death. Additionally, the authors noted deficiencies in several pDC antiviral functions, including reduced innate sensing due to decreased expression of *TLR7* and *DHX36*, as well as diminished antiviral effector functions and cytotoxicity [77].

In contrast to patients with moderate COVID-19 symptoms, conventional dendritic cells (cDCs) from individuals with severe illness displayed a diminished IFN signature and reduced MHC-II expression, despite the activation of pro-inflammatory pathways. A deeper investigation revealed that, in severe COVID-19 cases, there was a correlation between this decline and reduced levels of the MHC-II regulators RFX5, RFXANK, and CIITA. Viral interference with the antigen presentation capacity of DCs may contribute to the worsening of the disease in severe COVID-19 [77].

### 3.3. Natural Killer Cells and SARS-CoV-2

Natural killer (NK) cells play a crucial role in recognizing and destroying abnormal cells, including those infected by viruses. NK cells can be sub-grouped based on the expression levels of the CD56 and CD16 cell surface molecules. Specifically, CD56^dim^ CD16^+^ NK cells play a crucial role in antiviral host defense through cell-mediated cytotoxicity, while CD56^bright^ and CD56^dim^ CD16^dim^ NK cells contribute to antiviral immunity through IFN-γ and TNF-α production [78].

Patients with COVID-19 that develop lymphopenia show a reduction in the number not only of T cells but also of NK cells. The reduction in the count of natural killer (NK) cells, observed post-SARS-CoV-2 infection, may result from both cell death and the redistribution of cells to infected sites. This implies that NK cells could exit the bloodstream, relocate to the lungs, and play a role in virus clearance. However, this migration may also lead to local inflammation and tissue damage [79,80]. NK cells derived from COVID-19 patients, besides being reduced in number, also exhibit limited anti-viral activity and are characterized by an increased expression of the NK inhibitory marker NKG2A, which limits the production of effector molecules, such as CD107a, IFN-γ, IL-2, granzyme B, and TNF-α [81].

NK cells from patients with COVID-19 also activate a profibrotic transcriptional program, consisting of an upregulation of the *AREG, DUSP2, ZFP36L2*, and *TSC22D3* genes, which may promote fibrotic lung disease in patients with severe disease [82].

## 4. Innate Immunity and COVID-19 Vaccines

To control the spread of the COVID-19 pandemic, various vaccines have been developed to produce neutralizing antibodies specific to the S protein. Currently approved COVID-19 vaccines can be divided into four main categories: (1) RNA vaccines (e.g., Moderna’s Spikevax mRNA-1273 and Pfizer-BioNTech’s Comirnaty BNT162b2); (2) vector-based vaccines (e.g., AstraZeneca’s Vaxzevria, Covishield ChAdOx1, and Johnson & Johnson-Janssen’s Ad26.COV2.S); (3) protein subunit-based vaccines (e.g., Novavax’s Nuvaxovid and Covovax NVX-CoV2373); and (4) inactivated whole virus vaccines (e.g, Sinopharm’s Covilo, Sinovac’s CoronaVac, and Bharat Biotech’s Covaxin) (https://covid19.trackvaccines.org/agency/who/, accessed on 1 November 2023).

The first two categories include vaccines that do not physically contain the SARS-CoV-2 S protein but have the genetic information required for its production, such as mRNA and DNA vaccines. The third category is, instead, made of vaccines physically containing the SARS-CoV-2 S protein, which can be delivered in different forms and with added adjuvants. The fourth category consists of whole SARS-CoV-2 virus particles grown in cell culture that are chemically inactivated.

In addition to carrying the microbial antigen or the genetic information coding for it, which defines its specificity, a vaccine must be immunogenic, meaning that it has to be capable of efficiently activating the innate immune system in order to trigger an effective adaptive immune response and the consequent generation of immunological memory. The main difference between the four vaccine types in terms of their immunogenicity is that RNA vaccines and vector-based vaccines intrinsically and efficiently stimulate innate immune responses, and are indeed referred to as “self-adjuvanted” [83,84], while protein subunit-based and inactivated whole virus vaccines do not, and hence require additional adjuvant molecules.

All vaccine types are very efficient in preventing COVID-19, especially in protecting against severe disease and death (https://covid19.trackvaccines.org/agency/who/, accessed on 1 December 2023).

### 4.1. Differences in Innate Immune Responses Induced by Different Vaccines

COVID-19 mRNA vaccines contain a functional mRNA that is translated by the host translational machinery into the SARS-CoV-2 S protein. In this context, the genetic content of the vaccine provides both the information to produce the antigen and the adjuvant activity, since viral mRNA is recognized by the innate immune system and activates it. Rather, the mRNA contained in the vaccine has specific nucleotide modifications, namely cytosine, adenine, and uridine methylations, aimed at reducing the recognition by TLRs and RLRs to avoid an excessive innate immune response that, besides provoking reactogenicity to vaccination, could also restrict the translation of the encoded S protein [85,86], thus compromising the adaptive immune response to the antigen.

In addition to its genetic content, lipid nanoparticles (LNPs), which are used as a vaccine carrier, also contribute to the immunogenicity of mRNA vaccines. LNPs protect the mRNA, help with its entry into cells, and allow it to be delivered efficiently to the lymphatic system, specifically to the lymph nodes (LNs). LNPs play a role in eliciting inflammatory responses triggered by vaccines and serve as adjuvants to enhance adaptive immune responses [84].

**Figure 2:**
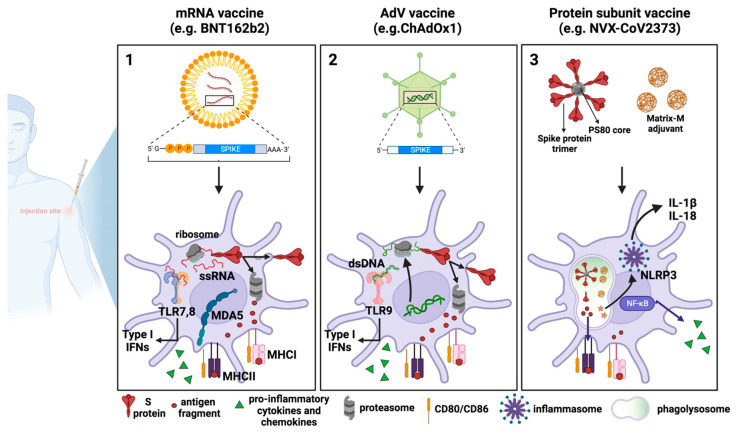
**COVID-19 vaccines: processing by dendritic cells and innate immune response activation**. Schematic representation of the mechanisms of action of the three major vaccine platforms against SARS-CoV-2: (**1**) mRNA-based vaccines, in which an mRNA molecule encoding for the SARS-CoV-2 S protein is encapsulated in lipid nanoparticles; (**2**) AdV vaccines, in which the SARS-CoV-2 S protein is encoded by a DNA molecule embedded in an AdV capsid; and (**3**) recombinant protein-based vaccines in which SARS-CoV-2 S proteins are assembled around nanoparticles to resemble the actual virus structure.

Upon entry into host cells, mRNA is detected by innate sensors, including TLR3, TLR7, and TLR8 in the endosome, and RIG-I and MDA-5 in the cytosol, leading to type-I IFN production [84] (Figure 2).

Compared to mRNA-based vaccines, Adenovirus Vector (AdV)-based DNA vaccines require a more complex pathway to reach the synthesis of functional mRNA and the production of the native S protein. An intranuclear passage of adenoviral DNA is required for transcription and RNA processing. This added complexity, combined with the nature of the vaccine, can lead to heterogeneous immune reactions, which can be attributed, for example, to the adenovirus type or to vector-specific genetic modifications.

The strategy used for current AdV-based COVID-19 vaccines is the replacement of the early region 1 (E1) adenoviral gene with the full-length SARS-CoV-2 S gene and the deletion of the E3 region. The deletion of the E1 gene, in addition to enabling the insertion of the S gene, is essential to abolish the replication of the viral vector, making the vaccine safe for the recipient. Deletion of the E3 gene, which is involved in evading host immunity but is not essential for virus production, is planned, along with that of the E1 region (E1/E3 deletion), to accommodate larger recombinant genes, such as SARS-CoV-2 S [87,88].

Innate immune responses triggered by AdV-based vaccines are different from those induced by mRNA vaccines since the DNA is detected by different PRRs. In particular, AdVs expose PAMPs sensed by TLR2 and TLR4 at the plasma membrane level, and by TLR9, which is located endosomally. Furthermore, the viral DNA can be detected following endosomal rupture via cytosolic DNA sensors like cGAS and the inflammasome, which promote the secretion of type-I IFNs [89] (Figure 2).

Among the approved SARS-CoV-2 recombinant protein subunit-based vaccines, the most widely used is NVX-CoV2373. It comprises the trimeric full-length SARS-CoV-2 S, which is generated as a recombinant protein in Sf9 cells through a baculovirus expression system. The SARS-CoV-2 S trimer is arranged around a polysorbate 80 (PS80)-based detergent mixed with the saponin-based adjuvant Matrix M [90]. At the site of injection and at the draining lymph nodes (dLNs), Matrix M triggers a rapid activation of innate immune cells, including APCs. The consequent cytokine release from APCs recruits additional innate immune cells, causing a cascading local immune response. Matrix M also induces the activation of NLRP3 inflammasome, which leads to the release of IL-1β and IL-18 and the production and secretion of other proinflammatory cytokines [91,92] (Figure 2).

Inactivated vaccines are derived from the complete SARS-CoV-2 virus, which has been rendered inactive through exposure to physical or chemical inactivating agents like UV rays, formalin, or formaldehyde administration [93]. Like SARS-CoV-2 recombinant protein subunit-based vaccines, inactivated whole virus vaccines must be combined with adjuvants to be effective and able to boost their immunogenicity. For example, the adjuvant used in Covaxin is a TLR7/8 agonist. TLR7 and TLR8 agonists potentiate the Th1 but suppress the Th2 immune response, an event which is beneficial for COVID-19 vaccines. TLR recognition in the innate cell population has also been linked to the generation of early type-I IFN, which promotes viral clearance and pro-inflammatory cytokine generation [94].

In contrast to the initial three vaccine categories where the S protein serves as the sole immunogen, inactivated vaccines elicit more extensive immune responses because of the presence of additional immunogenic proteins, such as the M, N, and E proteins. This results in a lower S-specific T cell response but is accompanied by a broader polyclonal T cell response, with T cells also being specific for other viral epitopes [95].

### 4.2. Innate and Adaptive Immune Response Cross-Talk upon COVID-19 Vaccination

Although the initial phase of the immune response to a vaccine involves the innate immune system, the effective immunization of the host requires the proper stimulation of the humoral and cellular adaptive immune system to induce the production of neutralizing antibodies on the one side and the differentiation of memory B cells and T cells on the other.

Professional APCs, such as DCs, bridge the two arms of the immune system by presenting the vaccine-associated antigen to naïve CD4^+^ and CD8^+^ T cells onto MHC-II and MHC-I molecules, respectively. Migratory DCs, which capture the antigen in the periphery and then migrate to the LNs, and LN-resident DCs, which uptake soluble antigen arrived into the LNs from the afferent lymphatics, induce protective immunity by priming antigen-specific naïve CD4^+^ helper T cells and CD8^+^ cytotoxic T cells. Certain CD4^+^ T cells with specificity for the antigen undergo differentiation into follicular T helper cells (Tfh). These Tfh cells play a role in facilitating the differentiation of B cells into high-affinity antibody-secreting plasma cells and memory B cells. This process leads to the production of specific neutralizing antibodies against the virus, establishing immune memory. Consequently, this protection helps prevent the individual from developing the disease and, in some instances, from acquiring the infection [96].

Both mRNA and AdV vaccines require multiple doses to gain optimal protection. More pronounced vaccine-associated inflammatory states have been associated with vaccine boosters compared to the first doses. This is a result of short-term “trained immunity” of innate cells (e.g., macrophages) and of the activation of memory T and B cells generated by the primary immune response [97]. The resulting production of type-I IFN amplifies T cell memory and promotes B cell differentiation and survival, contributing to the generation of long-lasting immunological memory.

In contrast to mRNA vaccines, during an AdV-based vaccination, pre-existing immunity to the vector may limit the ability to deliver genetic material to host cells, thus reducing the vaccine’s efficacy [98]. To overcome this issue, vectors from alternative AdV serotypes with low prevalence in the population have been developed, and different AdV vectors can be used for the first and the second dose of the vaccine. This was the strategy used, for example, in the original vaccination protocol with the Sputnik V vaccine that consisted of a two-dose regimen with two different human AdVAdV26 and AdV5. To mitigate the issue of vector-specific pre-existing immunity, AdV from other species such as chimpanzees, cattle, and pigs have been also used as candidates for vaccine development [99].

In contrast to mRNA and AdV vaccines, which lead to the intracellular production of the antigen, in protein subunit-based vaccines the antigen is taken up by APCs from the extracellular space. In this context, the activation of CD8^+^ T cells mostly relies on the cross-presentation capacity of APCs; this is the process by which an extracellular antigen, which would be normally loaded onto MHC-II, is presented by MHC-I molecules. Therefore, while antibody production and CD4^+^ T cell responses induced by protein-subunit-based vaccines are comparable to those of mRNA and AdV vaccines, the frequency of S-specific CD8^+^ T cells is lower [100]. Nonetheless, the orchestrated humoral and cellular immune response elicited by protein subunit-based vaccines is sufficient to efficiently protect individuals from COVID-19, especially from its severe forms [101].

Regarding the cell-mediated adaptive immune response, in the case of NVX-CoV2373, the MatrixM adjuvant has been shown to induce a preferential polarization of CD4^+^ T cells toward a Th1 response [102], preventing the immune response from veering toward Th2-like responses, which is undesirable for host defense against SARS-CoV-2 [103,104].

All three types of vaccines work similarly at the cellular level, as they all are endocytically internalized by DCs either at the site of injection or following trafficking to the dLNs. The vaccine-derived SARS-CoV-2 S protein is then degraded by the immunoproteasome into peptides that will be presented as antigens by the MHC class I. Furthermore, antigens released extracellularly can be phagocytosed by professional APCs, processed, and presented by MHC class II. What differs between vaccines is how the SARS-CoV-2 S protein is produced or processed. (1) In mRNA vaccines, after endosomal escape, mRNA can enter the cytosol and be immediately translated by the host cell translational machinery, in turn producing high levels of S protein. (2) Once the AdV escapes the endosome, the partially disassembled AdV capsids can reach the nucleus where the S transgene can be transcribed. After being exported into the cytoplasm, SARS-CoV-2 S mRNA can be translated into protein. (3) In the case of recombinant protein-based vaccines, for example, in NVX-CoV2373, the MatrixM adjuvant and SARS-CoV-2 S protein are internalized by host cells and are directly processed by phagolysosomes and by proteasome for antigen presentation. MatrixM induces the upregulation of co-stimulatory and MHC molecules, thus promoting the presentation of antigenic peptides. Due to their intrinsic adjuvant activity, mRNA and AdV vaccines are also able to activate innate sensors, which produce type-I IFNs, pro-inflammatory cytokines, and chemokines. Such sensors comprise TLR7, TLR8 and MDA5, which detect RNA and TLR9, which detects double-stranded DNA. Concerning recombinant protein-based vaccines, the addition of the MatrixM adjuvant in the formulation facilitates both the recruitment and activation of innate immune cells at the site of injection and the likely activation of the NLRP3 inflammasome, which mediates the maturation and secretion of the pro-inflammatory cytokines IL-1β and IL-18 through caspase-1 activation. (This figure was created with BioRender.com).

## 5. Concluding Remarks and Future Perspectives

A meticulous control of innate immune responses is critical for clearing pathogens, and a dysregulated innate immunity can lead to tissue damage and cause disease.

The pathophysiology of COVID-19 depends not only on the titer of infecting viral particle, but also on the degree of dysfunction of innate and adaptive immune responses. Uncoordinated immune responses in patients with severe COVID-19 cause a delay in the clearance of the virus, over-inflammation, and tissue damage, which are events that are not limited to the lungs but can spread throughout the body, leading to multi-organ failure [105,106]. This uncoordinated response mainly results from the ability of SARS-CoV-2 to evade the type-I IFN antiviral innate response [107] and from prolonged lymphopenia [108]. Subsequently, the uncontrolled accumulation of SARS-CoV-2 viral particles causes abnormal activation and recruitment of myeloid cells and an overt proinflammatory response, which contributes to immune pathology [109]. Indeed, patients with severe COVID-19 have increased levels of circulating inflammatory cytokines, which are associated with acute lung damage [110]. Given the direct cross-talk between innate and adaptive immunity, a dysfunctional innate immune response is also related to deficient adaptive immunity. The two main branches of adaptive immunity, the cell-mediated and humoral responses, were indeed found to be generally both deregulated upon SARS-CoV-2 infection in patients with COVID-19, causing dysfunctional helper, effector, and cytotoxic T cell activation as well as deregulated immunological memory and antibody production [111,112].

In this review, however, we explored the fundamental questions exclusively surrounding the innate immune response to SARS-CoV-2. We examined the current mechanisms of virus identification by PRRs, particularly those elicited by TLRs, RLRs, and NLRs, and also explored PRRs roles in facilitating effective innate immunity against COVID-19. During SARS-CoV-2 infection, signaling through such receptors has to be finely regulated as overstimulation might hyperactivate the innate immune system, harming the host through tissue damage and/or systemic inflammation.

In addition, we discussed the activation of innate immunity following SARS-CoV-2 vaccination, comparing the vaccine platforms that are currently approved and in use.

Overall, we can state that different types of SARS-CoV-2 vaccines can trigger, even if by different means, the innate immune response. Future studies are, however, mandatory to fully establish their impact on innate immune system activation.

Besides their efficacy, anti-COVID-19 vaccines have been reported to induce a strong proinflammatory response or autoimmune reactions. In rare cases, these reactions have resulted in serious adverse events, such as myocarditis and pericarditis, and, in the worst cases, even death [113,114]. These effects might be attributed to vaccine intrinsic adjuvanticity, especially for the novel platforms used in the design of these vaccines (mRNA, adenovirus-based vaccines), as well as to nonspecific immune responses or molecular mimicry events. Such effects can be exacerbated by individual comorbidities or by coincidental events that are not strictly related to the vaccine itself. These worst-case scenarios have contributed to heightening the skepticism already present regarding vaccine efficacy, leading to speculation that the increased mortality observed between 2020 and 2022 was actually correlated with COVID-19 vaccination rather than SARS-CoV-2 infection [115,116].

Despite the possibility that these rare and extreme events can actually occur, regulatory agencies strongly recommend COVID-19 vaccination as the best way to protect against serious illness, as the benefits outweigh its known and potential risks, especially in vulnerable individuals (https://www.cdc.gov/coronavirus/2019-ncov/vaccines/vaccine-benefits.html, accessed on 1 December 2023). It is of utmost importance that the CDC, the U.S. Food and Drug Administration (FDA), the European Medicines Agency (EMA), and other federal agencies continue to monitor the safety of COVID-19 vaccines and share their findings with the public as they become available.

Another important subject of reflection is the potential contribution of trained immunity in the protection against COVID-19. This term refers to an immunological process in which innate immune cells undergo long-term epigenetic and functional changes after infections or vaccinations. Certain types of approved vaccines, especially those based on live attenuated microorganisms (e.g., BCG vaccination) [117,118], were reported to induce broad heterologous protection against SARS-CoV-2 infection or decrease the clinical severity of COVID-19 and overall mortality [119]. Although the results on the benefits of trained immunity from approved vaccines remain inconclusive, efforts should be made to develop better-trained immunity-inducing vaccines as components of pandemic preparedness plans for the future. These vaccines should be able to harness the entire potential of the immune system to protect individuals against new and dangerous pathogens.

## Data Availability

Not applicable.
